# Comment on “Quantitative MRI Evaluation of Coracohumeral Ligament and Inferior Glenohumeral Capsule Thickening in Adhesive Capsulitis: Correlation with Range of Motion and Edema Patterns”

**DOI:** 10.5152/eurasianjmed.2026.261409

**Published:** 2026-05-11

**Authors:** Barış Acar

**Affiliations:** Department of Orthopaedic and Traumatology, İstanbul Education and Research Hospital, İstanbul, Türkiye

Dear Editor,

I read with interest the article titled “Quantitative MRI Evaluation of Coracohumeral Ligament and Inferior Glenohumeral Capsule Thickening in Adhesive Capsulitis: Correlation with Range of Motion and Edema Patterns.” The authors should be commended for attempting to standardize magnetic resonance imaging (MRI) measurements in adhesive capsulitis (AC), a condition in which imaging findings are often described qualitatively despite well-recognized structural alterations.^1^

Previous MRI studies have consistently demonstrated thickening of the coracohumeral ligament (CHL) and capsular hypertrophy at the axillary recess as characteristic features of AC.^2^^,^^3^ In addition, the fibroinflammatory progression of AC, initially described by Neviaser, provides a biological basis for correlating capsular thickening and edema with clinical restriction.^4^ In this regard, the authors’ effort to correlate CHL and inferior glenohumeral capsule thickness with range of motion is clinically meaningful and aligns with the pathophysiologic framework of the disease.

However, several points merit further discussion. First, although quantitative thresholds are proposed, the reproducibility of these measurements is not fully clarified. Coracohumeral ligament thickness measurements may vary depending on slice orientation, shoulder rotation, and field strength, as well as observer-dependent factors. These potential sources of variability highlight the importance of standardized imaging planes and consistent shoulder positioning to improve measurement reproducibility.^2^ Second, AC is widely regarded as a clinical diagnosis and MRI findings may overlap with other causes of shoulder stiffness, including rotator cuff pathology or post-traumatic capsular changes.^5^ A clearer description of exclusion criteria and potential confounders would strengthen the diagnostic specificity of the imaging parameters presented.

Furthermore, while edema patterns are interpreted as markers of inflammatory activity, the cross-sectional design limits conclusions regarding disease stage. Longitudinal evaluation would be necessary to confirm whether signal changes truly correspond to the painful “freezing” phase and whether they predict treatment response.

Despite these considerations, the study represents an important step toward objective—MRI-based assessment in AC. With further validation and the adoption of standardized measurement protocols, quantitative capsuloligamentous parameters may not only improve diagnostic accuracy but also contribute to disease staging and support more individualized treatment strategies in differentiating inflammatory and fibrotic phases of the condition.

## Data Availability

The data that support the findings of this study are available on request from the corresponding author.
